# Investigation of the Relationship Between IL-17, IL-27, IL-2 Blood Levels in Spontaneous Abortion and Healthy Pregnant Women

**DOI:** 10.3390/life15030326

**Published:** 2025-02-20

**Authors:** Gurkan Ozbey, Elif Seren Tanriverdi, Ayberk Cakir, Ercan Yilmaz

**Affiliations:** 1 Department of Obstetrics and Gynecology, Faculty of Medicine, Adıyaman University, 02040 Adıyaman, Türkiye; 2 Department of Medical Microbiology, Faculty of Medicine, Inonu University, 44000 Malatya, Türkiye; seren.tanriverdi@inonu.edu.tr; 3 Gynecology and Obstetrics Clinic, Acıbadem University Atakent Hospital, 34638 Istanbul, Turkey; drayberkcakir@gmail.com; 4 Department of Obstetrics and Gynecology, Faculty of Medicine, Inonu University, 44210 Malatya, Türkiye; ercan.yilmaz@inonu.edu.tr

**Keywords:** Thl cell, Th2 cell, pregnancy, IL-2, IL-17, spontaneous abortion

## Abstract

Background: Cytokines are essential for regulating immune cell activity during pregnancy. Research shows that CD4+ T-cells exhibit specific cytokine secretion patterns, resulting in polarized immune responses. This study aims to compare the gene expression levels of Th1, Th2, and Th17 cytokines in women with normal pregnancies versus those with a history of recurrent spontaneous abortion (RSA). Methods: In this case-control study, 20 patients with RSA within 24 h of their last abortion were compared to 20 pregnant women with no history of abortion (Control Group). Cytokine levels of IL-2, IL-17, and IL-27 were quantified using real-time polymerase chain reaction (RT-PCR). Results: Overall cytokine levels were similar between the groups, but the cytokine levels in both groups were generally similar. However, higher IL-17 and IL-2 levels were observed in the healthy pregnancy group (*p* = 0.006 and *p* = 0.001, respectively). Elevated IL-17 and IL-27 levels were observed in healthy pregnancies, whereas lower levels were seen shortly after a miscarriage. IL-27 levels were significantly higher in women with recurrent abortions compared to those with healthy pregnancies (*p* < 0.001). Conclusions: Elevated IL-2 levels may be a risk factor for RSA. Consistent with recent studies, our findings emphasize the role of IL-17 and IL-27 as crucial regulatory cytokines for maintaining a successful pregnancy.

## 1. Introduction

Pregnancy involves complex interactions between the mother and fetus, requiring immune tolerance, hormonal balance, and genetic and environmental factors. Key processes such as angiogenesis and endocrine regulation are essential for fetal development. Although reproductive immunology has been studied, its full role in pregnancy remains incompletely understood [1,2]. The maternal immune system adapts to accept the fetus, but this can sometimes lead to complications like pregnancy loss, preterm birth, and recurrent spontaneous abortion (RSA) [3,4]. Spontaneous abortions occur in 10–15% of pregnancies, with approximately 1% of women experiencing recurrent cases [1,2,3,4]. RSA is linked to genetic abnormalities, environmental toxins, hormonal imbalances, and infections [5]. Despite the fact that chromosomal abnormalities are often responsible for spontaneous abortions, RSA remains unexplained in 50% of cases, suggesting a potential role of immune factors [6]. Risk factors for RSA include the number of pregnancies and maternal age [5,6].

The immune system is crucial during pregnancy for maintaining tolerance and preventing fetal rejection. Natural killer (NK) cells in the uterine decidua are essential for a successful pregnancy [7]. Proper preconception care and immune balance are necessary for a healthy pregnancy, as immune cells and cytokines influence embryo implantation and fetal development [8,9]. NK cells, marked by CD56 and lacking CD3, constitute about 10% of peripheral blood lymphocytes and migrate to the endometrium and decidua, where they interact with integrin β2a1 and ICAM-1 on endothelial cells [8,10]. Although their precise role is not fully understood, NK cells, which constitute about 70% of endometrial leukocytes during the luteal phase, are vital for implantation, with disruptions potentially causing pregnancy loss [8,10]. T-helper (Th) cells, categorized into Th1 and Th2 types, regulate immune responses during pregnancy. Th1 cytokines (e.g., IFN-γ, TNF-α, IL-2) support cellular immunity but can negatively affect pregnancy, while Th2 cytokines generally offer protective effects [8,9].

IL-2 enhances NK cell activity and proliferation [11,12]. Research indicates that a Th2 response is typical in healthy pregnancies, while a Th1 response is linked to recurrent pregnancy loss (RPL). Cytokines from Th2 cells generally support pregnancy, whereas those from Th1 and Th17 cells can be detrimental. Pro-inflammatory cytokines like IL-1, TNF-α, IL-6, and IL-17 negatively impact pregnancy. IL-17, produced by Th17 cells, aids in rejecting conceptus antigens and promotes new blood vessel formation [9,13,14,15,16,17,18,19]. In the maternal-fetal interface, decidual cells release CCL2 to attract Th17 cells, which then secrete IL-17, preventing trophoblast cell apoptosis and encouraging their growth. IL-17 can also exacerbate autoimmune diseases such as multiple sclerosis and rheumatoid arthritis. The balance between Th1 and Th2 cytokines is crucial, with Th1 responses linked to complications and Th2 responses associated with successful pregnancies [20]. The Th1/Th2 model has now expanded to include Th17 cells, which may play a significant role in pregnancy maintenance by regulating implantation and fetal development [21]. IL-27, from the IL-12 family, is involved in inflammatory and autoimmune conditions [22]. It may reduce inflammation, but the IL-27-964 A > G polymorphism is associated with higher risk of spontaneous abortion. IL-27, produced by various immune cells, lowers pro-inflammatory cytokine levels and promotes Th1 cell differentiation [23]. IL-27 may impact pregnancy complications like preeclampsia and recurrent miscarriage by affecting inflammation in fetal membranes. High IL-27 levels are linked to low IL-17A levels, as IL-27 inhibits IL-17 production and reduces Th17 differentiation, potentially mitigating IL-17’s negative effects [24].

In addition to immune factors, recent studies have highlighted the importance of oxidative stress and efferocytosis in pregnancy complications. The interplay between immune responses and oxidative stress has been shown to contribute to the pathophysiology of RSA. Xanthine oxidase, a key enzyme in the production of reactive oxygen species, has been implicated in oxidative stress during pregnancy [25,26,27]. Furthermore, efferocytosis, the process of clearing apoptotic cells by macrophages, is essential for maintaining immune tolerance and preventing excessive inflammation during pregnancy. Dysfunctional efferocytosis has been linked to pregnancy complications, including RSA [26]. The role of xanthine oxidase and CD93 in regulating immune responses and oxidative stress during pregnancy is an area of ongoing research, and their involvement in pregnancy complications warrants further investigation [27,28].

Cytokines like IL-17, IL-27, and IL-2 are crucial in regulating immune system functions. IL-17 is associated with inflammation and autoimmune disorders, indicating an imbalanced immune response [6]. IL-27 is important for immune regulation and response modulation [7]. IL-2 supports cellular immune functions and maintains immune balance [8]. Variations in these cytokine levels between women with spontaneous abortion and healthy pregnancies may exist, but their impact on pregnancy outcomes is not fully understood. Research suggests that increased NK cell activity, as well as elevated IL-2 and IL-27 levels, may serve as risk factors for spontaneous abortion. This study aims to compare the gene expression levels of IL-2, IL-17, and IL-27 cytokines between women with normal pregnancies and those with spontaneous abortions.

This study includes 20 healthy pregnant women (ages 20–30) with no history of miscarriage and 20 women (ages 18–30) who have experienced spontaneous abortion. Blood samples were collected during the first trimester from the miscarriage group and during the second or third trimester from the healthy pregnancy group. Participants with autoimmune disorders, genetic anomalies, inflammatory diseases, or other systemic conditions were excluded. Approximately 8 mL of blood was collected from each participant. For the miscarriage group, samples were obtained within 24 h post-abortion at Muş State Hospital, centrifuged, and plasma was stored at −80 °C. Gene expression levels of IL-2, IL-17, and IL-27 cytokines were compared between the two groups using real-time PCR at the Medical Microbiology Department of Inonü University. This aided in investigating the impact of these cytokines on pregnancy health and in providing fundamental data on the etiology of the condition.

## 2. Materials and Methods

### 2.1. Study Design and Participant Grouping

This study compares two groups: Control Group: 20 healthy pregnant women aged 21–26 with no history of abortion, recruited during the second or third trimester. Patient Group (Group I): 20 women aged 24–29 who have experienced spontaneous abortion.

Blood samples (approximately 8 mL) were collected from all participants. For the patient group, samples were collected during the first trimester and within 24 h of the miscarriage. For the control group, samples were collected during the second or third trimester. The first trimester was selected for the RSA group, as most spontaneous abortions occur during this period, allowing for an investigation of immune system dynamics early in pregnancy. This enables a more accurate assessment of cytokine level differences between women with spontaneous abortion and those without. For the control group, the second and third trimesters were chosen, as these periods are associated with greater immune stability and tolerance in healthy pregnancies. By examining immune responses during these stages, the study aims to compare the immune status of women with normal pregnancies to those with recurrent spontaneous abortion (RSA), providing insights into the immune mechanisms that may influence pregnancy outcomes. Blood samples were obtained from the Obstetrics and Gynecology Department of Muş State Hospital, facilitating a comparative analysis of immune markers between the two groups. Blood samples were centrifuged to collect plasma, which was stored at −80 °C at the Obstetrics and Gynecology Clinic of Muş State Hospital. Later, gene expression levels of IL-2, IL-17, and IL-27 cytokines were analyzed at the Department of Medical Microbiology, Inonu University using real-time PCR (RT-PCR). RNA was isolated using the Qiasymphony RNA kit (Qiagen, Hilden, Germany). RNA quantity and purity were assessed with the MaestroNano Micro-Volume Spectrophotometer (MN-913, Maestrogen, Hsinchu City, Taiwan). cDNA synthesis was performed with the Quantitect Reverse Transcription Kit (Qiagen, Hilden, Germany). Subsequently, gene expression analysis of IL-2, IL-17, and IL-27 cytokines was performed using the Quantitect SYBR Green PCR kit (Qiagen, Hilden, Germany) with the RotorGeneQ real-time PCR system (Qiagen, Hilden, Germany). Expression levels were normalized to the GAPDH housekeeping gene.

### 2.2. Ethic Statement

Individuals with autoimmune conditions, genetic abnormalities, inflammatory diseases, or other systemic disorders were not included in this study. Informed consent was obtained from all participants after thoroughly explaining this study’s objectives and procedures. This study adhered to the Declaration of Helsinki and was approved by the local ethics review board (approval number: 2023/50).

### 2.3. Clinical Measurement

#### 2.3.1. RNA Extraction/cDNA Synthesis Protocol

All RNA isolation procedures were carried out in the Laboratory of Inonu University, Department of Medical Microbiology. Total RNA was extracted using the RNeasy^®^ Plus Mini Kit (Qiagen, Germantown, MD, USA) following the manufacturer’s instructions. cDNA synthesis was conducted with the RT^2^ First Strand cDNA Synthesis Kit (Qiagen) using 2 µg of total RNA in a Labcycler Thermal Cycler (SenSoquest, Göttingen, Germany). For RT-PCR, a 91 µL dilution of molecular grade water was used as the template. Following the RNA isolation processes, the RNA amounts obtained were checked for RNA concentrations and purity levels at a wave length of 260/280 nm on the Nanodrop (MaestroNano Micro-Volume Spectrophotometer (MN-913, Maestrogen, Taiwan)) device. According to the recommendation of the relevant kit company, RNA samples were stored at −80 °C until gene expression.

#### 2.3.2. Real-Time PCR Procedure

Real-time PCR analysis was performed using the Qiagen Rotorgene Q (Qiagen, Hilden) model. SYBR Green Master Mix (Qiagen) was used for the reactions, which were conducted in a total volume of 25 µL on 72 discs. Each reaction included 12.5 µL of SYBR Green PCR Master Mix (Amplicon, Odense, Denmark), 1 µL of each forward and reverse primer, 5 µL of undiluted cDNA, and 6.5 µL of nuclease-free water [29,30]. The PCR conditions were optimized as follows: initial denaturation at 95 °C for 5 min, followed by 40 cycles of denaturation at 95 °C for 15 s, annealing at 65 °C for 15 s, and extension at 72 °C for 45 s, with temperature transition rates of 20 °C/s. Each sample was analyzed in duplicate. The GAPDH gene served as an internal control for normalization.

Relative expression levels of cytokines (IL-17, IL-27, IL-2) and GAPDH were calculated using the 2^−ΔΔCt^ method. Primer sequences for human IL-17, IL-27, IL-2, and GAPDH are listed in Table 1 [29,30]. ABI Sequence Detection System software version 1.0 (PE Applied Biosystems, Warrington, UK) was used to determine the cycle number at which fluorescence emission crossed the threshold Ct value. The comparative Ct method, using arithmetic formulas, was used for the relative quantification of target mRNA with the Relative Expression Software Tool (REST©) 2009 [21]. Amplification efficiency was compared between the target gene and the reference control, GAPDH, for the 2^−ΔΔCt^ calculation.

### 2.4. Statistical Analysis

The data were analyzed using IBM SPSS v.26 (IBM Corp, Armonk, NY, USA) and R 4.0.5. Descriptive statistics are presented as mean and standard deviation or median and mode for numerical variables, and as number and percentage (%) for categorical variables. The Shapiro–Wilk test, Kolmogorov–Smirnov test, and residual analysis were used to determine whether the data were normally distributed. The assumption of sphericity was tested. In the analysis of independent group differences, the independent sample *t*-test was used when parametric test assumptions were given, and the Mann–Whitney U-test was used when assumptions were not given. The significance level was set at *p* < 0.05. Sample size was determined using the G*Power analysis system (version 3.1, Heinrich-Heine-University Düsseldorf, Düsseldorf, Germany). According to the sample size calculation, at least 40 individuals (spontaneous abortion patients = 20 and healthy pregnant women = 20) should be included for alpha 0.05 and 80% power.

## 3. Results

Table 2 contains a comparison of the demographic and baseline characteristics of spontaneous abortion patients and healthy pregnant women. Neither the spontaneous abortion patients nor the healthy pregnant women were smokers, and none had any additional diseases. Age, gravity, parity, abortion, smoking, presence of chronic disease and IL-2 were similar between the spontaneous abortion patients and healthy pregnant women (*p* > 0.05). Week of pregnancy, *GAPDH*, and IL-27 were significantly lower in spontaneous abortion patients than healthy pregnant women (*p* < 0.05). IL-17 was significantly higher in spontaneous abortion patients than healthy pregnant women (*p* < 0.05) (Table 2).

**Figure 1:**
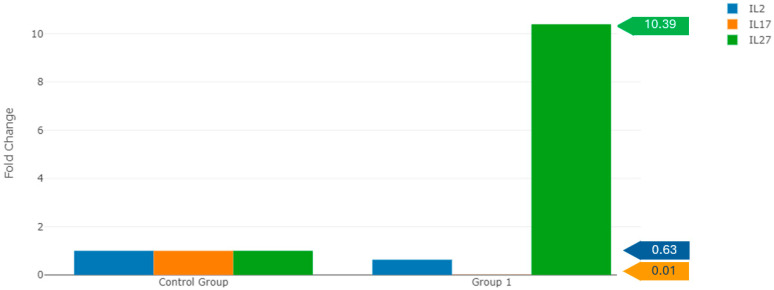
Comparison of fold changes in expression levels of IL-2, IL-17, and IL-27 between spontaneous abortion patients and healthy pregnant women. Group I: Patients with recurrent spontaneous abortion (RSA), Control group: Healthy pregnant women with no history of abortion.

The study observed that IL-27 expression was 10.39 times higher in patients experiencing spontaneous abortion compared to healthy pregnant women (*p* < 0.000001). This finding suggests that IL-27 may be significantly involved in the pathophysiology of spontaneous abortion. IL-27 is known as a cytokine that regulates immune responses, and its elevated expression could indicate an enhanced maternal immune reaction against the fetus, potentially leading to pregnancy loss. Furthermore, this study found that the expression levels of IL-2 and IL-17 were reduced by 0.63-fold and 0.01-fold, respectively, in pregnant women with spontaneous abortion (Figure 1). IL-2 is a critical cytokine that supports the proliferation and function of T cells, and its reduced levels might suggest that the immune system is inadequately protecting the fetus. Similarly, low levels of IL-17 may indicate a weakened defense against infections or an imbalance in immune regulation.

**Figure 2:**
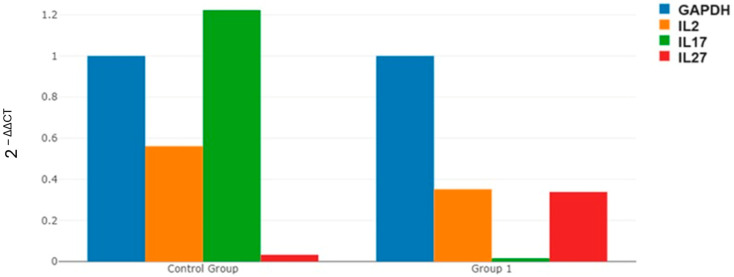
Comparison of 2^−ΔΔCT^ expression levels of IL2, IL17, and IL27 between spontaneous abortion patients and healthy pregnant women. Group I Patients with recurrent spontaneous abortion (RSA). Control group: Healthy pregnant women with no history of abortion.

There was no statistically significant difference in the 2^−ΔΔCT^ value for GAPDH expression levels between the spontaneous abortion patients and the healthy pregnant women (*p* = 1.000). This result indicates that GAPDH is expressed at similar levels in both the patient and control groups, making it a suitable reference gene for normalization in this study. The lack of a statistically significant change in GAPDH expression supports its reliability as a reference gene for the normalization process. It was observed that the 2^−ΔΔCT^ in the expression level of IL-2 in spontaneous abortion patients decreased compared to healthy pregnant women, but this decrease was not statistically significant (*p* = 0.509). It was observed that IL-17 decreased in a statistically significant manner in spontaneous abortion patients compared to healthy pregnant women (*p* = 0.039). It was observed that the 2^−ΔΔCT^ in the expression level of IL-27 in spontaneous abortion patients increased compared to healthy pregnant women, but this decrease was not statistically significant (*p* = 0.855) (Figure 2, Table 3).

**Figure 3:**
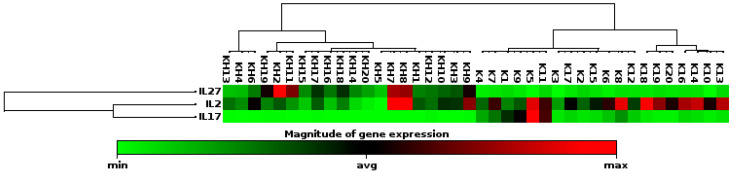
Comparison of expression levels of IL-2, IL-17, and IL-27 between spontaneous abortion patients and healthy pregnant women.

In patients with spontaneous abortion, a 10.39-fold increase in IL-27 expression was observed compared to healthy pregnant women (Figure 3). This finding shows that IL-27 is strongly upregulated (increased) in patients with spontaneous abortions and suggests that this cytokine may be associated with inflammatory or immune responses related to spontaneous abortion.

## 4. Discussion

The Th1/Th2 paradigm not only elucidates the fundamental mechanisms underlying the protection and progression of various immunopathological conditions but also serves as a foundation for developing novel therapeutic approaches [31]. Understanding the interactions between antigen profiles and the host genetic background not only enables the control of infectious diseases but also supports the development of new therapeutic strategies for managing immunopathological reactions. This study elucidates the role of gene expression levels in cases of spontaneous abortion and provides initial data for the organization of future treatment protocols aimed at improving quality of life. The findings from this study provide fundamental information about a potential immune regulator necessary for maintaining a normal pregnancy.

Th cells are classified into four types: Th0, Th1, Th2, and Th17. Naive Th0 cells differentiate into Th1, Th2, or Th17 cells in response to antigens, depending on the cytokine environment. Th1 cells produce IFN-γ, IL-2, and TNF-β, which activate macrophages and CTLs, leading to a cell-mediated immune response. Th2 cells produce IL-4, IL-5, IL-6, IL-9, IL-10, and IL-13, promoting B cell activation and antibody production. IFN-γ inhibits Th2 and Th17 cell development, while IL-10 prevents Th1 cells from producing IFN-γ. IL-4 may inhibit Th1 production and Th17 differentiation. Immune responses vary based on the pathogen, with cell-mediated responses targeting intracellular pathogens and antibody responses targeting extracellular ones. IL-27, which inhibits Th17 differentiation, is located on chromosome 16p11 and consists of five exons, highlighting its critical role in T-cell development [32,33,34,35].

The findings of this study reveal that IL-2, IL-27, and IL-17 levels differ between RSA and healthy pregnancies. Our study provides important data on the relationship between these cytokines and pregnancy health, as well as recurrent miscarriages.

In this study, when comparing the demographic and basic characteristics of the included spontaneous abortion patients and healthy pregnant women (Table 2), the median age of the spontaneous abortion patients was found to be 27.50 years [27,28,29,30,31,32], while the median age of the healthy pregnant women was 24 years (21.50–26.50). This difference was not statistically significant (*p* = 0.055). The lack of statistical significance in age difference suggests that age may not be a determining factor in spontaneous abortion. However, the slightly higher median age in the spontaneous abortion group may warrant consideration of age-related risk factors. The median gravidity value was 3 (2–5) for spontaneous abortion patients and 2 (2–4) for healthy pregnant women. This difference was not statistically significant (*p* = 0.378). The lack of statistical significance in the difference in gravidity suggests that spontaneous abortion may be independent of the number of pregnancies. This indicates that the number of pregnancies may not directly affect the risk of spontaneous abortion. The median parity value was 2 (1–3) for spontaneous abortion patients and 1 (1–3) for healthy pregnant women, and this difference was not statistically significant (*p* = 0.657). The lack of a significant difference in parity indicates that the number of births may not be a determining factor in the risk of spontaneous abortion. This result suggests that parity might be unrelated to spontaneous abortion or that other factors may have a more pronounced effect. When evaluating the history of abortions in both groups, the median abortion value was 0 (0–1) for spontaneous abortion patients and 0 (0–0) for healthy pregnant women, with no significant difference (*p* = 0.095). The lack of a significant difference in abortion history may indicate that most of the current spontaneous abortion cases are not related to previous abortions or that the current abortions are due to factors other than cumulative effects. None of the participants in either group use tobacco (*p* = 1.000). The absence of differences in tobacco use suggests that smoking is not a significant factor affecting spontaneous abortion in this study or that tobacco use is equally low in both groups. There are no chronic diseases in either group (*p* = 1.000). The absence of chronic diseases in both groups may exclude their effects on spontaneous abortion and indicate that the condition may result from other factors. The median gestational age of spontaneous abortion patients was 7 weeks (6–7), while for healthy pregnant women it was 37 weeks (31.50–39), and this difference is statistically significant (*p* < 0.001 *). The fact that spontaneous abortion patients are at an earlier gestational age indicates that this group experienced abortion during the early stages of pregnancy and that gestational age is an effective factor in the risk of spontaneous abortion. When evaluating the levels of IL-2, IL-17, and IL-27 cytokines according to gestational weeks between the groups: IL-2 levels in spontaneous abortion patients were measured at 18.31 ± 0.74 and in healthy pregnant women at 18.02 ± 0.58, with no significant difference found (*p* = 0.187). The lack of a significant difference in IL-2 levels might suggest that IL-2 is not associated with spontaneous abortion or that larger sample sizes might be needed to uncover interesting results. However, the IL-17 level was 22.76 ± 1.65 in spontaneous abortion patients and 16.90 ± 1.53 in healthy pregnant women, and this difference was statistically significant (*p* < 0.001). Elevated IL-17 levels suggest that there may be an increased inflammatory response in patients with spontaneous abortion, and this cytokine could play a role in the pathogenesis of spontaneous abortion. This situation indicates that high levels of IL-17 might be associated with immune system dysregulation and could contribute to pregnancy loss. Additionally, the median IL-27 level in spontaneous abortion patients was found to be 18.46 (18.14–18.76), while in healthy pregnant women it was 21.65 (21.35–21.91), with this difference also being statistically significant (*p* < 0.001). The significant decrease in IL-27 levels in spontaneous abortion patients may reflect the effects of this cytokine on immune response. Low levels of IL-27 could indicate a disruption in immune regulation or the effects of inflammation, which might serve as a biological marker for spontaneous abortion.

Recurrent miscarriages may be linked to proinflammatory cytokines transforming NK cells into cytotoxic lymphocytes, which can target and destroy trophoblast cells. Type 1 cytokines like TNF-α and IFN-γ can induce apoptosis in trophoblast cells, suppress growth-supporting cytokines from the uterine epithelium, and activate coagulation pathways, potentially leading to vasculitis and impaired maternal blood supply to the embryo [36,37]. Chaouat et al. found that injecting IL-2, TNF-α, and IFN-γ at specific levels could terminate a normal pregnancy [36]. Additionally, TNF-α may cause fetal expulsion due to uterine contractions or embryo necrosis [38]. It has been suggested that TNF-α, in combination with hormones, contributes to placental thrombosis, which may result in miscarriage; TNF-α levels rise at the onset of labor and during spontaneous abortion [38].

When assessing the fold changes in IL-2, IL-17, and IL-27 expression levels between spontaneous abortion patients and healthy pregnant women, this study found that IL-27 expression levels were 10.39 times higher in patients who experienced spontaneous abortion compared to healthy pregnant women (*p* < 0.000001). In women experiencing spontaneous abortion, IL-2 and IL-17 expression levels decreased by 0.63-fold and 0.01-fold, respectively (*p* = 0.006007 and *p* = 0.001464) (Figure 1). The decrease in the levels of these cytokines may indicate a disruption in the immune tolerance necessary for the continuation of pregnancy. IL-2 plays a critical role in T-cell proliferation and function, which is essential for maintaining immune balance during pregnancy. The decrease in IL-2 expression might suggest a reduction in protective immune response in patients with spontaneous abortion. IL-17 regulates inflammatory responses. The low levels of these cytokines may indicate a deficiency in the immune regulations necessary for maintaining pregnancy. IL-17 plays a role in promoting inflammation and providing protection against infections. The decrease in IL-17 levels in patients with spontaneous abortion might indicate reduced inflammatory responses, which could contribute to the pathogenesis of spontaneous abortion. The existing literature presents varying views on the role of IL-2 and IL-17 in pregnancy. Some studies suggest that low levels of these cytokines are associated with pregnancy losses, while others state that this association is not statistically significant [39,40,41,42]. While our findings suggest a significant reduction in IL-17 levels, potentially linked to spontaneous abortion (RSA), it is important to consider the conflicting results observed in the literature regarding the role of IL-17 in RSA. Some studies have proposed that IL-17 may support immune defense against pathogens during pregnancy by promoting inflammation and immune responses, potentially serving as a protective factor for fetal tolerance. In contrast, other studies indicate that excessive IL-17 production may lead to inflammation and immune dysregulation, contributing to pregnancy loss. These inconsistencies may stem from factors such as sample size, methodological differences, or the influence of other inflammatory mediators that modulate the effects of IL-17. For instance, while our study observed reduced IL-17 levels in spontaneous abortion patients, IL-17 may interact with other cytokines or immune factors, modulating its role in pregnancy outcomes. Further investigation into these interactions and the independent effects of IL-17 on immune responses could provide additional insights into its role in RSA. The timing and duration of IL-17 production during pregnancy may also determine its impact. A decrease in IL-17 early in pregnancy may disrupt maternal-fetal immune tolerance, while an increase in later stages could represent a compensatory response to inflammatory stimuli, such as infections or immune system activation. Understanding the optimal timing of IL-17 production during pregnancy could help clarify its complex role in RSA. Given these complexities, larger and more diverse cohorts are needed in future studies to reconcile the differing perspectives on IL-17’s role in pregnancy. Additionally, a more detailed analysis of the cytokine environment is crucial in evaluating IL-17 as a therapeutic target for preventing RSA. IL-2 plays a critical role in T-cell proliferation and function, which is important for maintaining immune balance during pregnancy. The decrease in IL-2 expression might suggest a reduction in protective immune responses in patients with spontaneous abortion. However, since the change is not statistically significant, its impact on pregnancy outcomes remains unclear and requires further investigation.

In our study, IL-27 levels were found to be 10.39 times higher in patients with recurrent miscarriages, with this increase being statistically significant (*p* < 0.000001). The increase in IL-27 suggests that it might play a significant role in the pathogenesis of recurrent miscarriages and could be used as a potential biomarker. Additionally, the effects of IL-27 on immune responses should be carefully examined in both fetal and maternal contexts. IL-27 is an important cytokine that regulates immune responses and inflammation, and elevated levels of IL-27 in recurrent miscarriages may indicate enhanced inflammatory responses or impaired fetal tolerance. Increased levels support the idea that IL-27 is related to the immunological processes in miscarriages [34,35]. This suggests that IL-27 may play a significant role in spontaneous abortions. Our study provides findings that support these discussions and offer new perspectives. In particular, our finding that IL-27 could emerge as an important biomarker in recurrent miscarriages is a rare approach in the current literature. In conclusion, the fold changes in IL-2, IL-17, and IL-27 expression levels between spontaneous abortion patients and healthy pregnant women reveal possible alterations in immune regulation that might contribute to spontaneous abortion. The marked decrease in IL-17 and the substantial increase in IL-27 indicate that these cytokines may play pivotal roles in the immunological processes contributing to spontaneous abortion. Additionally, the changes in IL-2, IL-17, and IL-27 in recurrent miscarriages can contribute to our understanding of the roles of these cytokines in immune responses and inflammation processes. However, to determine the clinical significance of these findings and the precise roles of these cytokines in the pathogenesis of spontaneous abortion, larger and more detailed studies are needed. Furthermore, it is clear that further research is required to investigate the interactions of these cytokines and their relationships with other immune parameters.

In our study, when comparing the 2^−ΔΔCT^ expression levels of IL-2, IL-17, and IL-27 between patients with spontaneous abortion and healthy pregnant women, the expression of GAPDH did not show a statistically significant difference between the two groups (*p* = 1.000). This result indicates that GAPDH is expressed at similar levels in both groups, which confirms its suitability as a normalizing reference gene for this study. The lack of a statistically significant change in GAPDH expression supports its reliability as a reference gene in normalization processes. In patients with spontaneous abortion, the 2^−ΔΔCT^ value of IL-2 was decreased compared to healthy pregnant women, but this decrease was not statistically significant (*p* = 0.509). This may suggest that IL-2 could play a role in the pathophysiology of spontaneous abortion, but the data obtained in this study do not strongly support this relationship. IL-2 is an important cytokine associated with T cell activation and proliferation and may play a critical role in maintaining fetal tolerance during pregnancy [38]. However, the role of IL-2 in recurrent miscarriages is complex; some studies suggest that low levels of IL-2 may impair the immune system’s ability to support fetal tolerance, while others have not found this decrease to be statistically significant [36]. This discrepancy may be related to sample size and methodological characteristics of the studies.

A statistically significant decrease in IL-17 2^−ΔΔCT^ expression levels was observed in patients with spontaneous abortion compared to healthy pregnant women (*p* = 0.039). This reduction suggests that IL-17 plays a significant role in regulating immune responses and is strongly associated with spontaneous abortion. The decrease in IL-17 levels may indicate maternal-fetal immune dysregulation and an increased risk of miscarriage. Conversely, while IL-27 2^−ΔΔCT^ values were higher in spontaneous abortion patients compared to healthy pregnant women, this increase was not statistically significant (*p* = 0.855). While IL-27 may play a role in spontaneous abortion, the lack of statistical significance in its expression limits the strength of this association in the present study. In summary, the significant decrease in IL-17 may be considered a potential biomarker associated with spontaneous abortion, whereas changes in IL-2 and IL-27 were not statistically significant. These findings highlight the potential role of IL-17 in immune processes related to spontaneous abortion and suggest that further detailed investigation of this cytokine is warranted in future studies. The lack of statistical significance in changes observed in other genes suggests a need for larger sample sizes or additional studies. These findings provide important insights into the role of the immune system in the pathophysiology of spontaneous abortion; however, further research is required to strengthen these results. This analysis highlights the complexity of the immune response associated with spontaneous abortion and suggests that IL-17 may be a significant cytokine in this context, warranting further investigation.

In this study, it was observed that the 2^−ΔΔCT^ expression level of IL-2 in spontaneous abortion patients decreased compared to healthy pregnant women, but this decrease was not statistically significant. In contrast, IL-17 levels were significantly reduced in spontaneous abortion patients compared to healthy pregnant women, whereas IL-27 levels were significantly increased (Figure 2, Table 3). These findings indicate that, while some immune responses are hyperactive (IL-27) in women experiencing spontaneous abortion, others (IL-2 and IL-17) are insufficient. This imbalance could hinder the healthy development of the fetus and increase the risk of spontaneous abortion. Therefore, the regulation of these cytokines may be considered a potential therapeutic strategy to reduce the risk of spontaneous abortion. This study addresses an important gap by examining the differences in IL-2, IL-27, and IL-17 levels between recurrent miscarriage and healthy pregnancy in detail. Although various studies in the literature explore the role of these cytokines during pregnancy, there is limited information specifically on the association of IL-27 with recurrent miscarriage and its interactions with the other two cytokines.

Our study contributes to the literature as one of the first to reveal the significant increase of IL-27 in spontaneous abortions, highlighting its potential effects on fetal-maternal immune balance. Additionally, it demonstrated a decrease in IL-2 and IL-17 levels in spontaneous abortions, which may be associated with a disruption of immune tolerance. These findings provide crucial data for a better understanding of the immunological mechanisms underlying recurrent miscarriages and support the potential use of these cytokines as biomarkers. Although our study provides important insights, there are several limitations. First, the limited sample size may restrict the generalizability of our findings. Future large-scale studies involving diverse populations are essential to validate these findings. Second, methodological differences in cytokine level measurements may make the direct comparison of our results with other studies challenging. Additionally, this study did not investigate other immunological and genetic factors contributing to the pathogenesis of spontaneous abortion, which may prevent a complete understanding of the results. Despite these limitations, the uniqueness and contribution of our findings offer a new perspective on the immunological mechanisms of recurrent miscarriages and provide a solid foundation for future research. However, considering the study’s limitations, further research is needed to enhance the accuracy and generalizability of the findings.

Our findings suggest that elevated IL-27 levels may be a risk factor for recurrent spontaneous abortion (RSA). To support a normal pregnancy, it may be necessary to modify and/or down-regulate NK cell cytotoxicity.

## Figures and Tables

**Table 1:** The forward and reverse of primer sequences [30,31].

The sequences for IL-2;	
Forward	5′-TTTACATGCCCAAGAAGGCCA-3′ [30]
Reverse	5′-GCACTTCCTCCAGAGGTTTG-3′
The sequences for IL-17;	
Forward	5′-GGGCCTGGCTTCTGTCTGA-3′
Reverse	5′-AAGTTCGTTCTGCCCCATCA-3′ [30]
The sequences for GAPDH;	
Forward	5′-GCA CCG TCA AGG CTG AGA AC-3′ [31]
Reverse	5′-TGG TGA AGA CGC CAG TGG A-3′
The sequences for IL-27 *için primer dizileri;*	
Forward	5′-CTTGGCTGGCGTCTCAGCCT-3′ [31]
Reverse	5′-CGGAGAGCAGCTTCTCGGCG-3′

**Table 2:** Demographic and baseline characteristics of spontaneous abortion patients and healthy pregnant women.

	Spontaneous Abortion Patients (*n* = 20)		Healthy Pregnant Women (*n* = 20)		*p* Value
Age (years)	27.50 (24–29)		24 (21.50–26.50)		0.055
Gravity	3 (2–5)		2 (2–4)		0.378
Parity	2 (1–3)		1 (1–3)		0.657
Abortion	0 (0–1)		0 (0–0)		0.095
Smoking (%)	Yes	0	Yes	0	1.000
	No	100	No	100	
Presence of chronic disease (%)	Yes	0	Yes	0	1.000
	No	100	No	100	
Week of pregnancy	7 (6,7)		37 (31.50–39)		<0.001 *
*GAPDH*	16.80 ± 0.44		17.19 ± 0.63		0.029 *
IL-2	18.31 ± 0.74		18.02 ± 0.58		0.187
IL-17	22.76 ± 1.65		16.90 ± 1.53		<0.001 *
IL-27	18.46 (18.14–18.76)		21.65 (21.35–21.91)		<0.001 *

*: significant *p* value less than 0.05. Significant values are presented in bold.

**Table 3:** Comparison of 2^−ΔΔCT^ expression levels of IL2, IL17, and IL27 between spontaneous abortion patients and healthy pregnant women.

Gene Symbol	2^−ΔΔCT^		
	Control Group	Group 1	*p* Value
GAPDH	1.000000	1.000000	1.000
IL-2	0.560972	0.351476	0.509
IL-17	1.223488	0.016064	0.039 *
IL-27	0.032566	0.338329	0.855

*: significant *p* value less than 0.05. Significant values are presented in bold. Group I Patients with recurrent spontaneous abortion (RSA). Control group: Healthy pregnant women with no history of abortion.

## Data Availability

The data that support the findings of this study are available on request from the corresponding author. The data are not publicly available due to privacy or ethical restrictions.
